# Supplementary figures and images for: Evaluating place cell detection methods in Rats and Humans: Implications for cross-species spatial coding
Source: PLoS Comput Biol. 2026 May 26;22(5):e1013488. doi: 10.1371/journal.pcbi.1013488 (PMC13225663; doi:10.1371/journal.pcbi.1013488)

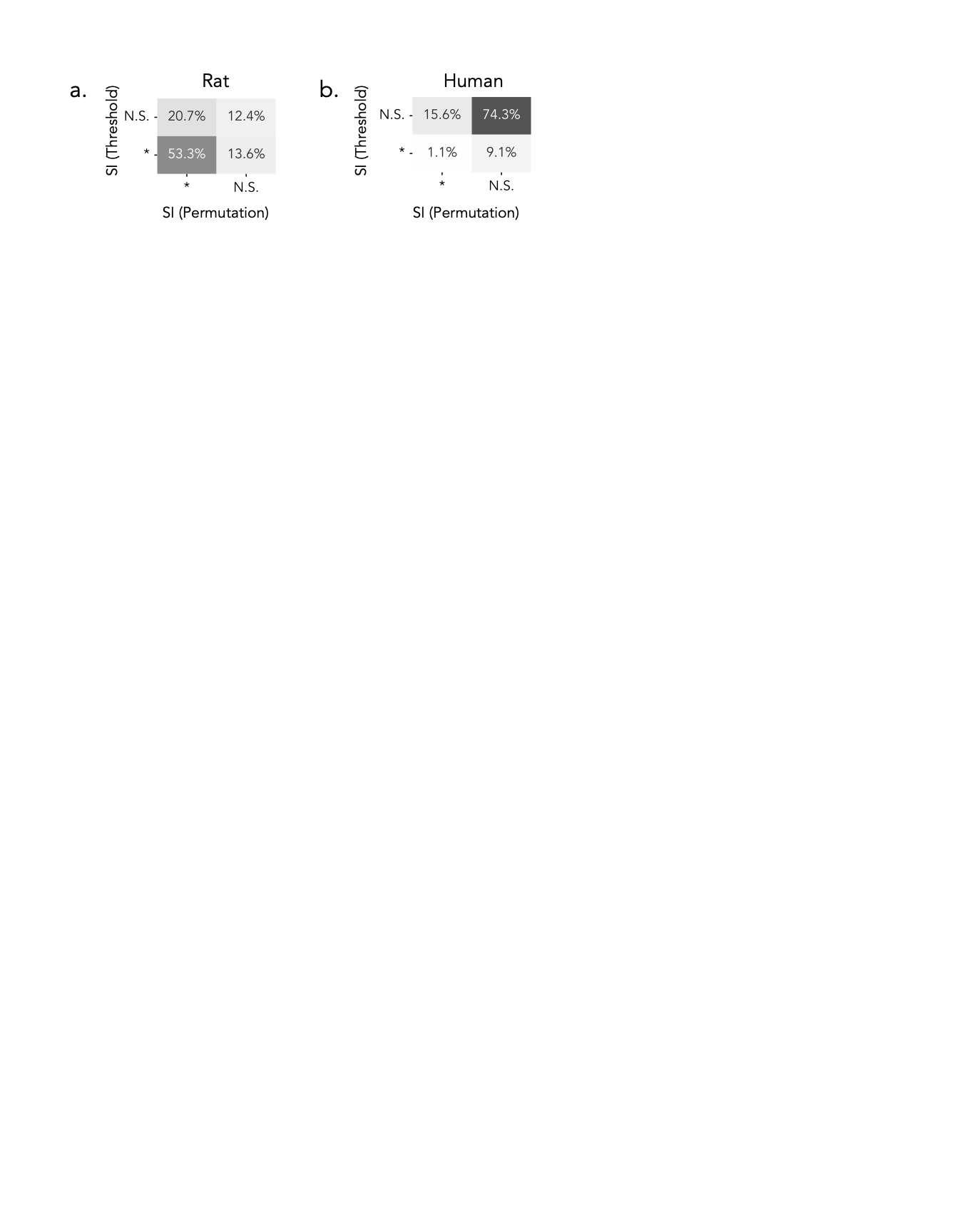

Supplement: S1 Fig — Each cell shows the percentage of neurons classified as significant (*) or non-significant (N.S.) a) Rats: Comparison of SI classifications using a fixed threshold (SI > 0.25) and permutation-based significance testing. b) Same comparison in human neurons. (TIFF) [file pcbi.1013488.s001.tiff]

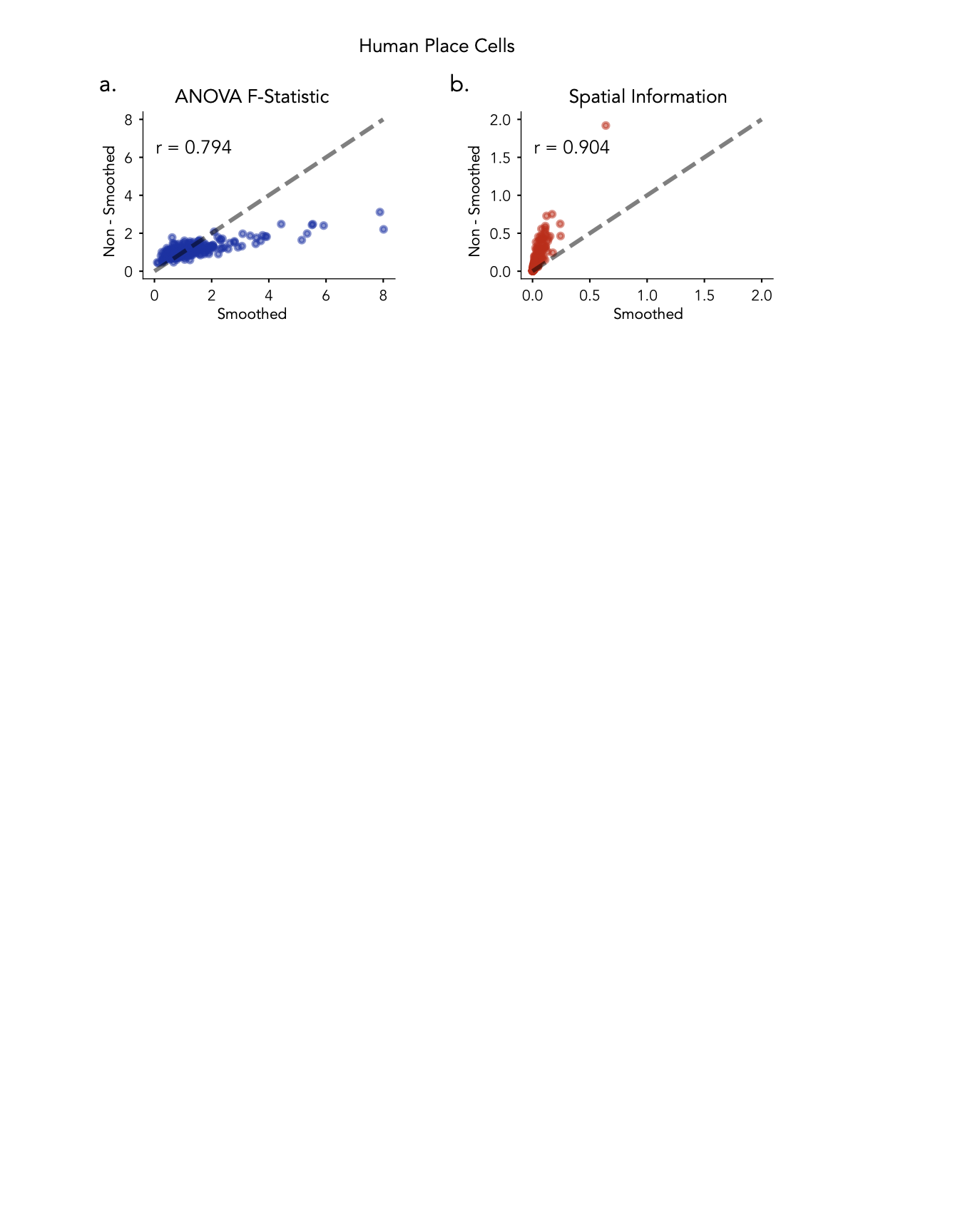

Supplement: S2 Fig — Each point represents a single neuron. a) ANOVA F-statistics computed from smoothed versus non-smoothed firing rate maps. b) Spatial information scores computed from smoothed versus non-smoothed data. (TIFF) [file pcbi.1013488.s002.tiff]

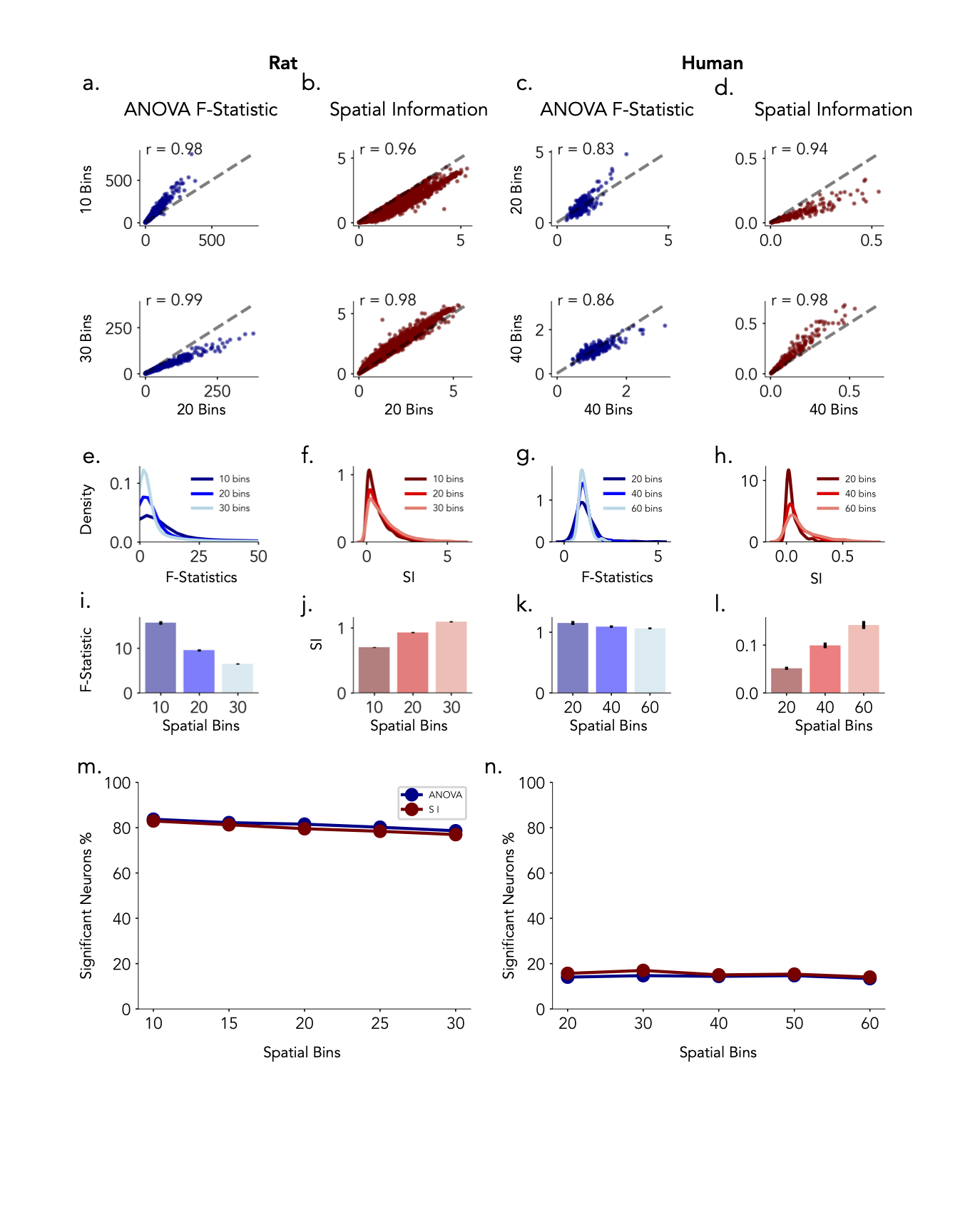

Supplement: S3 Fig — Rat data. a) ANOVA F-statistics comparisons: (Top) 20 vs. 10 spatial bins. (Bottom) 20 vs. 30 spatial bins. b) Spatial Information comparisons: (Top) 20 vs. 10 spatial bins. (Bottom) 20 vs. 30 spatial bins. Human data. c) ANOVA F-statistics comparisons: (Top) 40 vs. 20 spatial bins. (Bottom) 40 vs. 60 spatial bins d) Spatial Information (SI) comparisons: (Top) 40 vs. 20 spatial bins. (Bottom) 40 vs. 60 spatial bins. e–f) rat and g–h) human show the distributions of ANOVA F-statistics and Spatial Information across three spatial binning resolutions: 10, 20, 30 bins for rats and 20, 40, 60 bins for humans. i–j) rat and k–l) human summarize the average F-statistics and SI values for each binning resolution. m–n) show the percentage of significantly tuned neurons (y-axis) detected using ANOVA (blue) or SI (red) methods as a function of spatial binning (x-axis), for rats (m) and humans (n). (TIFF) [file pcbi.1013488.s003.tiff]

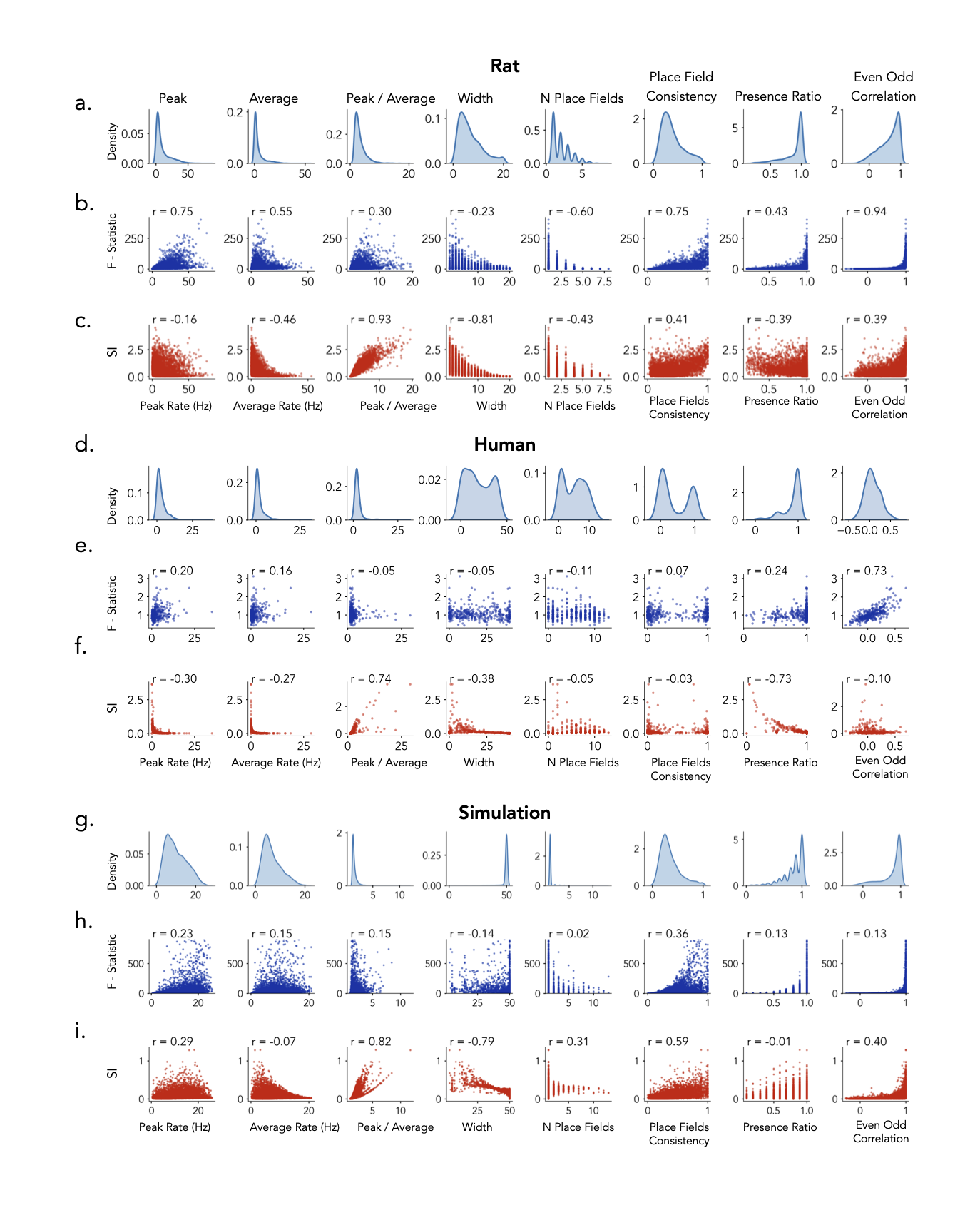

Supplement: S4 Fig — Panels show how two tuning metrics: ANOVA F-statistics and SI relate to eight neural features: peak firing rate, average firing rate, peak-to-average ratio, place field width, number of place fields, place field consistency, presence ratio, and even–odd correlation. a-c, show data from rat recordings: a) distributions of each feature. b) F-statistics vs. features. c) SI vs. features. d-f) Human neurons. g-i) Simulated neurons. Each scatter plot includes the Pearson correlation coefficient r, quantifying the strength of association between tuning metrics and neural features. (TIFF) [file pcbi.1013488.s004.tiff]

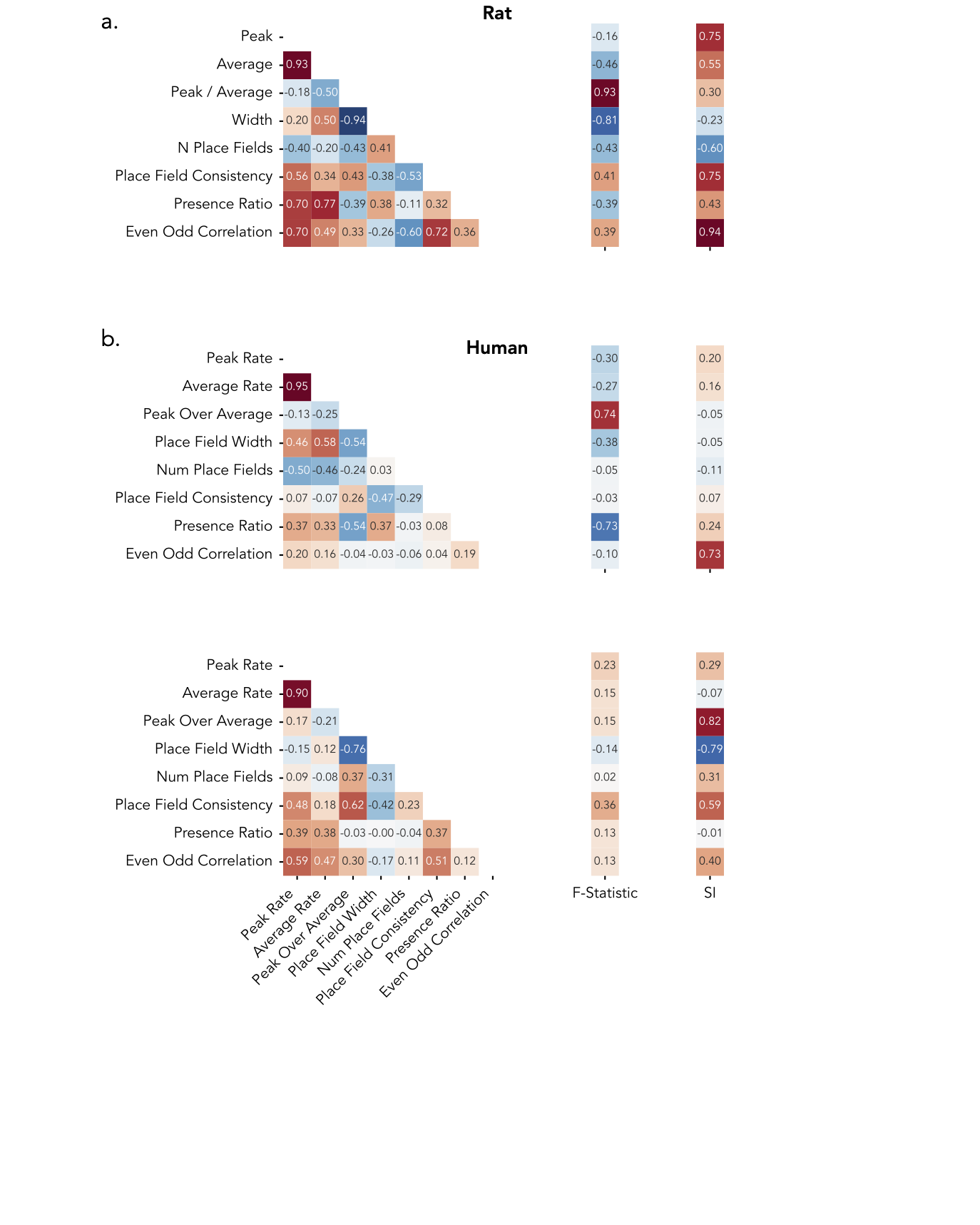

Supplement: S5 Fig — a) Rat dataset. b) Human dataset. c) Simulated dataset. Left, pairwise Pearson correlation coefficients between eight neural features: peak firing rate, average firing rate, peak-to-average ratio, place field width, number of place fields, place field consistency, presence ratio, and even–odd correlation. Middle, correlations between each feature and spatial information (SI). Right, correlations between each feature and ANOVA F-statistic. Color scale indicates the strength and direction of the correlation (red: positive; blue: negative). (TIFF) [file pcbi.1013488.s005.tiff]

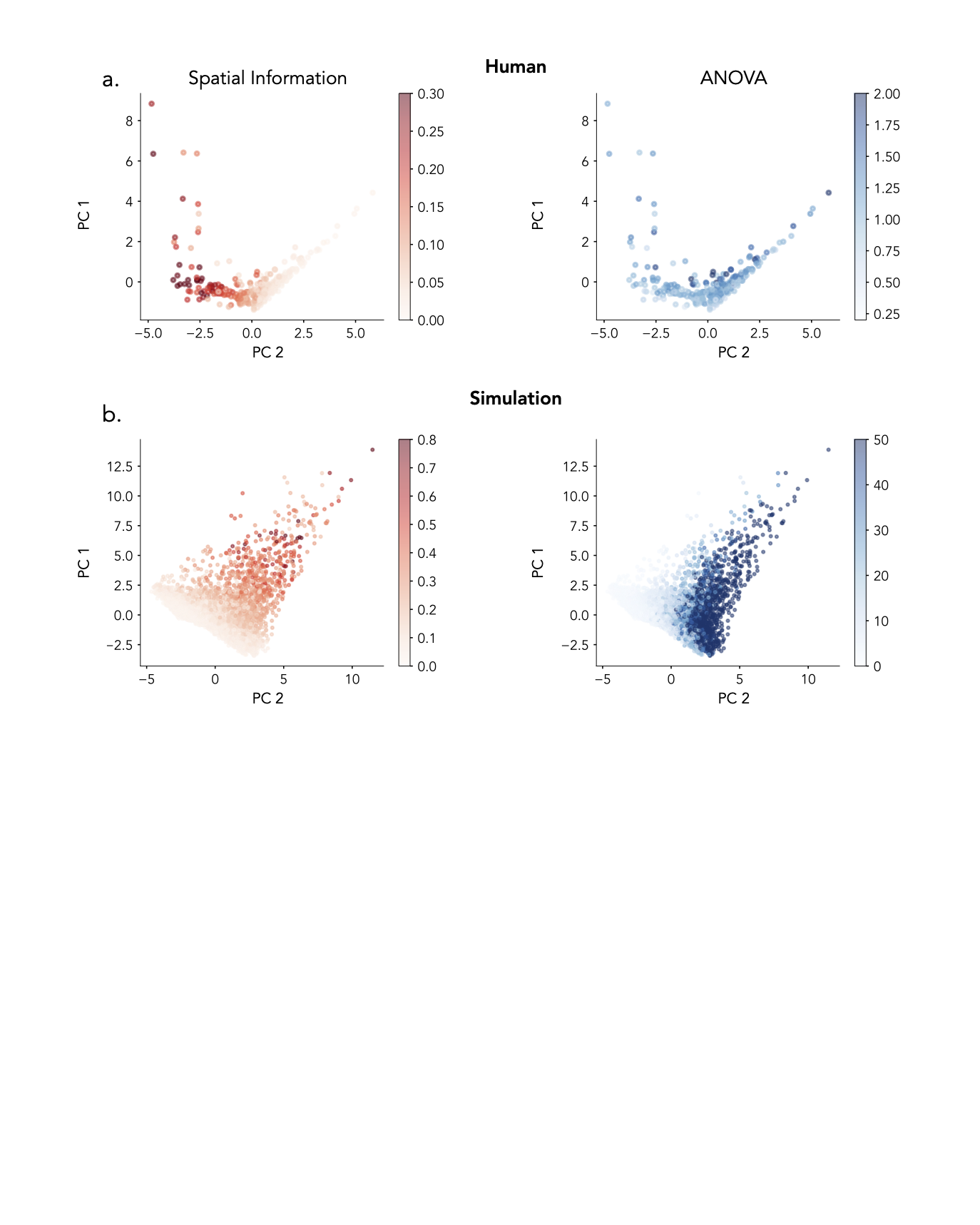

Supplement: S6 Fig — a) Human neurons projected onto the first two principal components, colored by spatial information (SI) and ANOVA F-statistics. b) Same as a), for simulated data. (TIFF) [file pcbi.1013488.s006.tiff]

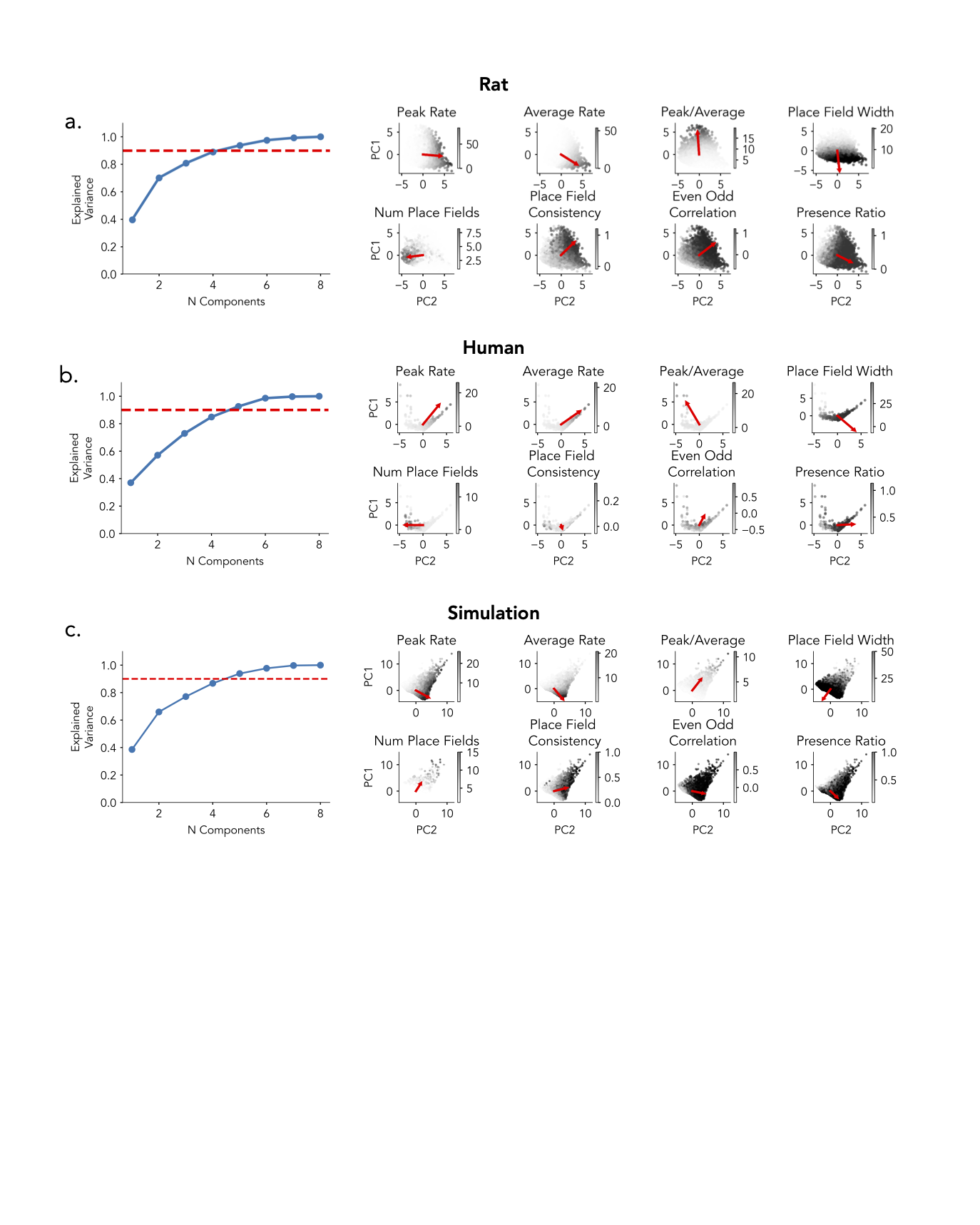

Supplement: S7 Fig — Principal component analysis (PCA) was performed on eight firing-related features for a) rat. b) human. c) simulated datasets. Left, variance explained by each principal component; blue dots indicate cumulative explained variance, and the red dashed line marks the 90% variance threshold. Right, biplots showing PCA projections of neurons onto the first two principal components (PC1 and PC2), with feature loadings (red arrows) overlaid. The direction and length of the red vectors indicate each feature’s contribution to the PCA axes. (TIFF) [file pcbi.1013488.s007.tiff]

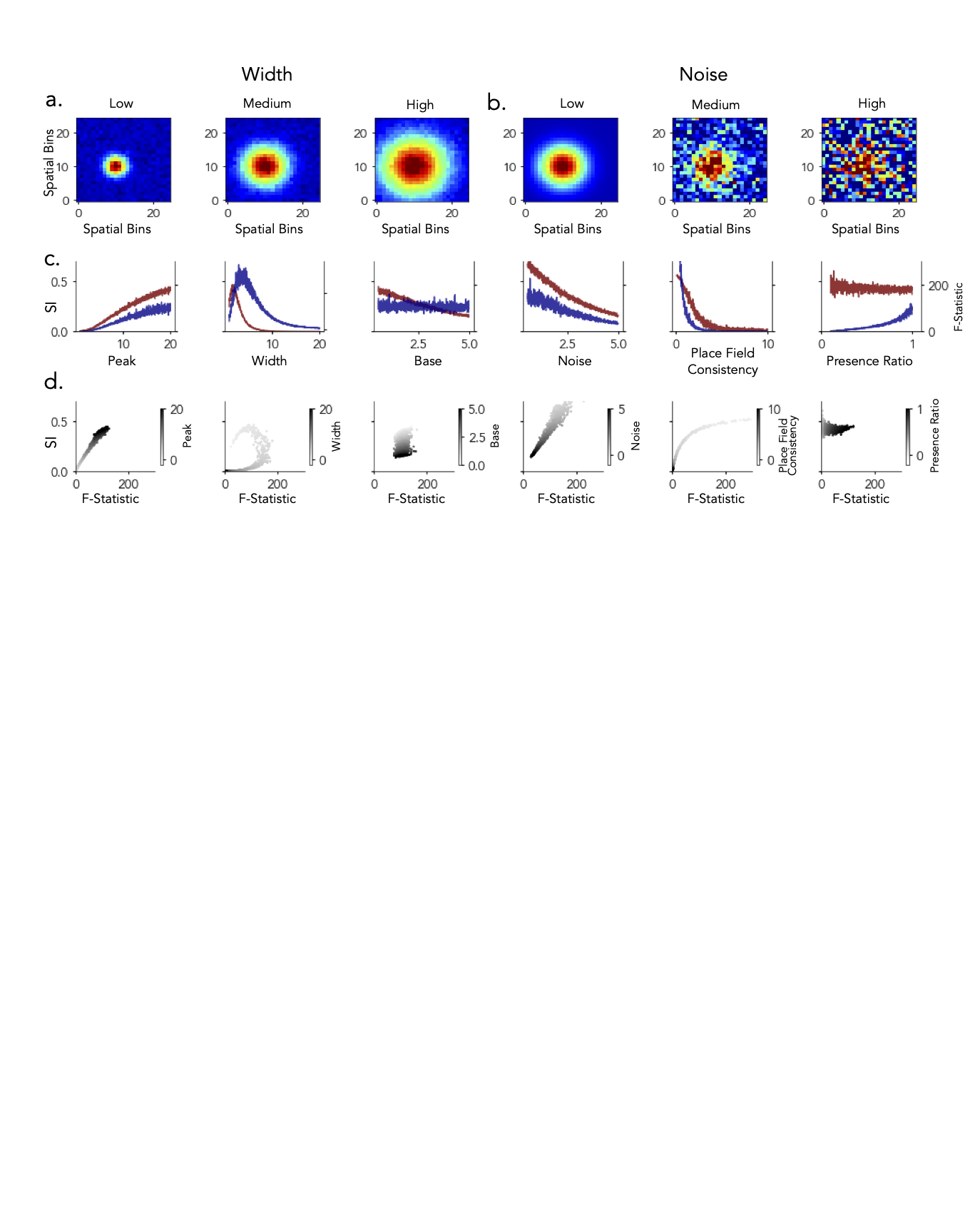

Supplement: S8 Fig — a) Simulated 2D firing rate maps illustrating variation across different width levels. b) Simulated 2D firing rate maps illustrating variation across different noise levels. c) Spatial information (SI, red) and ANOVA F-statistics (blue) as a function of each place field parameter in 2D environments. d) Joint distributions of SI (y-axis) and ANOVA F-statistics (x-axis), with grayscale indicating values of the corresponding parameter in 2D environments. (TIFF) [file pcbi.1013488.s008.tiff]
