# Supplementary material for: Evaluating place cell detection methods in Rats and Humans: Implications for cross-species spatial coding
Source: PLoS Comput Biol. 2026 May 26;22(5):e1013488. doi: 10.1371/journal.pcbi.1013488 (PMC13225663; doi:10.1371/journal.pcbi.1013488)
Supplement: Attachment — Submitted filename: PLDM-ResponseLetter.pdf [file pcbi.1013488.s009.pdf]

Manuscript Title: Evaluating Place Cell Detection Methods in Rats and Humans – Implications for Cross-Species Spatial Coding
Manuscript Number: *PCOMPBIOL-D-25-01752*
Journal: *PLOS Computational Biology*

Dear Editor and Reviewers,

We thank the Editor and Reviewers for their thoughtful and constructive feedback, and we appreciate the time and effort invested in evaluating our manuscript. Below, we provide a detailed point-by-point response, with reviewer comments in **black**, our responses in **blue**, and all corresponding changes marked in **red** in the revised manuscript. We hope these revisions address all major points raised by the reviewers and believe they have led to a strengthened manuscript.

Editor Comments:
In particular, the authors should ensure that their representation of the rodent place cell literature is accurate, consider extending their analyses to 2D firing rate maps, and include some consideration of more recent metrics based on information-theoretic measures, as further detailed in the reviewers' comments below. Finally, given that rodent electrophysiology studies of spatial responses are much more mature, they should endeavour to clarify whether the ANOVA is ever a useful metric for identifying and describing place cell firing patterns.

We appreciate the Editor's guidance and have revised the manuscript to address these broader concerns.

Representation of the rodent place-cell literature: We have reviewed and updated the Introduction and Discussion to provide a more accurate and comprehensive representation of rodent place-cell studies. We also revised Table 1 to better reflect current analytical practices (see Reviewer 2, Major Comment 1).

Extension to 2D firing-rate maps: We clarified in the discussion that ANOVA has been previously applied in both 1D and 2D human single-neuron studies, and we added simulations demonstrating that the comparative properties of SI and ANOVA extend to 2D environments (see Reviewer 1, Major Comment 2).

Consideration of recent information-theoretic metrics: We expanded the Discussion to address the use of more recent analytical approaches, including information-theoretic measures, and clarified how our findings relate to these methods (see Reviewer 1, Major Comment 3).

Clarifying the role of ANOVA in rodent place-cell analysis: In the revised text, we strengthened our manuscript by stating that ANOVA effectively identifies spatially modulated neurons in both rodent and human datasets, particularly neurons with weaker but consistent spatial modulation, such as those observed in the rodent ventral hippocampus and human single-neuron recordings. This overlapping population, which exhibits similar empirical properties, may be functionally meaningful across species, potentially supporting mixed selectivity and generalization (see Reviewer 1, Major Comment 5).

Journal Requirements:
Please note that we have addressed the journal Requirements in our resubmission.

Reviewer 1:
Zhang et al. compare two commonly used analytical approaches for identifying and characterizing spatially modulated neurons: spatial information content (SI) and analysis of variance (ANOVA), in both rodent and human hippocampal recordings. The authors also employ a combination of real neural data and simulated firing patterns to investigate how these methods vary in their sensitivity to firing field stability, spatial specificity, and overall detection rate. They further explore how analytical choices and thresholding criteria influence the classification of neurons as place cells, and how these methodological differences may affect cross-species comparisons. The topic addresses an important methodological gap in the field, as criteria for defining spatial cells are inconsistent across laboratories and species. The manuscript is well written, clearly illustrated, and makes a valuable contribution by encouraging a more principled approach to place cell classification. While there are no major analytical issues, and I agree with the authors' overall interpretation, I worry that the impact and general applicability of this work are overly specific.

Major comments
1. The introduction defines rat place cells as exhibiting "sharply localized and stable firing fields that tile the environment with high spatial precision." It would strengthen the manuscript to provide a clearer operational definition of a place cell, as interpretations may differ across fields, especially between rodent electrophysiology and human cognitive neuroscience. For example, how should cells that fire broadly but consistently across the environment be classified? Likewise, should spatially precise but unstable cells be considered place cells? Establishing a consistent definition at the outset would help contextualize the comparisons between analytical methods.

We thank the reviewer for highlighting the need for a clearer operational definition of a place cell. In this investigation, we are interested in place cells as broadly construed as neurons whose firing is indicative of the animal's spatial location, including both traditional place cells, characterized by sharply localized fields, and the broader yet consistent spatially modulated firing patterns observed across species, which may carry important functional significance(Introduction, lines 128-135). We further clarify how different analytical methods capture distinct properties of spatial tuning and how these insights help inform best practices for place-cell detection across species.

The revised section of the manuscript reads:

Place cells are typically defined as neurons that exhibit spatially selective firing when the animal occupies a specific location [2]. Here, we take a broad perspective on place cell activity, employing statistical detection methods that capture spatial tuning, including neurons with classical, sharply tuned, place fields, as well as more diverse spatial response patterns (e.g. responses that may have broader or more variable responses, as long as they are exhibit in a way that is captured by the detection methods). By using this approach, We hope to clarify how different analytical methods reflect the underlying properties of place cells, informing best practices for place-cell detection across species. To this end, we perform a systematic evaluation of place cell detection methods using both

2. The study focuses on recordings from linear track experiments, but most place cell studies are conducted in two-dimensional environments. It is unclear how well the ANOVA-based approach generalizes to two-dimensional data or to unstructured explorations, such as open-field foraging. Can the authors comment on whether ANOVA can be extended to such paradigms, and if not, whether this limits the method's practical utility for broader applications?

The revised section of the manuscript reads:

We thank the reviewer for raising this important point regarding the applicability of ANOVA-based place-cell identification in two-dimensional environments. In response, we have expanded both our analysis and discussion to provide a comprehensive approach to addressing this issue. First, we now explicitly note in the Discussion (Lines 489-500) that ANOVA-based approaches have been successfully applied in prior human navigation studies conducted in two-dimensional virtual environments, demonstrating that the method is not inherently restricted to one-dimensional trajectories.

Second, we performed additional simulations in 2D comparing spatial information (SI) and ANOVA F-statistics across different place-cell properties. Consistent with the 1D results, SI and ANOVA exhibit similar relative behavior in two dimensions. This outcome is expected, as both metrics operate over spatial bins and do not inherently differentiate firing patterns by dimensionality (e.g., 1D vs. 2D). These findings suggest that the comparative properties of SI and ANOVA described in the main text generalize to two-dimensional environments, as shown in the new supplemental (Fig. S8).

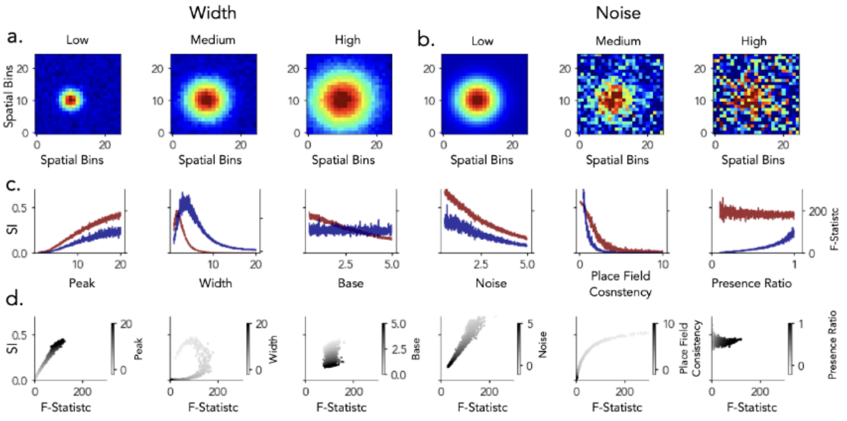

Figure S8: **Impact of place field features on spatial information and ANOVA statistics in 2D Environments.** a, Simulated 2D firing rate maps illustrating variation across different width levels. b, Simulated 2D firing rate maps illustrating variation across different noise levels. c, Spatial information (SI, red) and ANOVA F-statistics (blue) as a function of each place field parameter in 2D environments. c, Joint distributions of SI (y-axis) and ANOVA F-statistics (x-axis), with grayscale indicating values of the corresponding parameter in 2D environments.

3. The authors note that few studies have directly compared human and rodent hippocampal activity under comparable behavioral paradigms. However, in the present study, rodents actively explored a linear track, whereas humans were passively moved along a virtual track. Passive transport is known to disrupt spatial coding in rodents, which raises concerns about the comparability of the two datasets. This behavioral discrepancy may also explain the smaller proportion of human place cells observed and their reduced spatial specificity. The authors should address this limitation more explicitly and discuss its potential impact on the conclusions.

To address this point, we have added a dedicated section in the Discussion (see *Passive vs. Active Navigation: lines 568-577)* that explicitly acknowledges how behavioral differences may affect spatial coding and the comparability of human and rodent datasets. We also emphasize that future human studies employing active navigation or more immersive paradigms will be crucial for assessing the extent to which self-motion signals influence place-cell properties in humans.

The revised section of the manuscript reads:

Passive vs. Active Navigation

A related factor influencing the interpretation of place cell across-species is the distinction between passive and active navigation. Previous rodent work demonstrates that removing self-motion cues, such as through passive transport or head-fixed conditions, can weaken or destabilize spatial tuning and that visual input alone is insufficient to sustain robust place-cell firing during passive viewing [59, 60]. These findings suggest that the passive nature of the human navigation paradigm may contribute to the smaller proportion and broader tuning of spatially modulated neurons observed here. Nevertheless, future studies using actively controlled or more immersive navigation paradigms in humans will be important for determining the extent to which passive movement influences observed spatial coding patterns.

4. The field has begun to adopt more flexible analytical frameworks, such as generalized linear models (GLMs) that can account for collinearity and behavioral confounds, or information-theoretic measures like Kullback–Leibler divergence that are more robust to firing rate differences. Could the authors discuss how their findings might extend to or inform these more contemporary approaches? This would help situate the present work within the evolving methodological landscape.

As suggested, we have revised the manuscript to discuss how GLM-based approaches and KL divergence relate to our current analyses. We first clarify that GLM-based frameworks, similarly to ANOVA, offer flexible tools for modeling spatial firing while accounting for behavioral covariates and firing-rate differences; however, they typically require larger datasets, careful model specification, and substantial regularization, which can be challenging for human single-unit recordings with limited trial counts. In this setting, the ANOVA framework offers a constrained, interpretable, and data-efficient approach that is well-suited to our dataset. KL divergence, as a measure for comparing distributions, is, to our understanding, most commonly used to quantify changes in firing-rate distributions across task conditions.

Future studies could specify how KL divergence might be appropriately adapted for place-cell classification. This distinction is now made explicit in the revised manuscript (Lines 478 -387).

In Relation to Other Methods

In addition to SI and ANOVA, methods such as generalized linear models (GLMs) and information-theoretic measures provide alternative ways to characterize and compare spatial tuning. GLMs, conceptually similar to ANOVA, allow firing rates to be modeled as a function of multiple behavioral covariates, providing a flexible approach for handling correlated predictors, though they typically require larger datasets and careful regularization than is available in most current human single-neuron recordings[52]. KL-based measures quantify differences in firing-rate or population-activity distributions across task or environmental conditions [53, 54] and are better suited for assessing changes in the shape of firing distributions. These approaches illustrate the range of available methods for assessing spatial modulation across different datasets and analytical goals.

5. The finding that SI is more sensitive to spatial specificity while ANOVA is more sensitive to trial-to-trial stability is interesting, but perhaps unsurprising given the mathematical properties of the two metrics. SI inherently reflects spatial contrast and signal-to-noise ratio, whereas ANOVA quantifies variance across trials. The authors could strengthen their argument by clarifying what conceptual or practical insight emerges from demonstrating this distinction.

As suggested, we revised the manuscript (Lines 461–467) to clarify the conceptual and practical insights that emerge from this distinction. By applying both metrics to the same rodent and human datasets, we demonstrate that these differences result in variation in place-cell classification and impact the interpretation of spatial coding across species. Specifically, neurons with sharply localized but trial-variable firing can achieve high SI scores, whereas neurons with broader yet trial-consistent spatial firing patterns, commonly observed in rodent ventral hippocampus and human single-neuron recordings, are more likely to be identified by ANOVA-based criteria. These additions clarify how methodological choices emphasizing spatial contrast versus trial-level reliability can shape inferences about spatial modulation across species.

The revised section of the manuscript reads:

internally consistent. In summary, SI primarily captures field contrast whereas ANOVA F-statistics evaluate spatial differentiation and trial-level reliability, providing a complementary views on spatial coding. Neurons with sharply localized firing fields may achieve high spatial information scores even when their firing is highly variable across trials due to task-dependent modulation [51]. Neurons

24

with complex, broader yet trial-consistent spatial firing patterns, as commonly observed in the rodent ventral hippocampus and in human single-neuron recordings [35, 1], are more likely to be identified by ANOVA-based criteria.

Minor comments
1. Please include line numbers for ease of corrections.

The revised manuscript now includes line numbers to facilitate easier reference and correction.

2. The studies listed in Tables 1 and 2 should be cited in the reference list; otherwise, it is unclear which works the tables refer to.

All studies listed in Tables 1 and 2 have now been cited in the reference list, and the tables have been updated to ensure clear correspondence between each entry and its citation.

3. The rationale for using a spatial information cutoff of 0.25 should be clarified. The authors later refer to this as a “commonly used fixed cutoff,” but most rodent studies in Table 1 use 0.5. Since nearly all cells exceed significance at

this higher threshold (Fig. 2e), 0.25 seems unusually permissive and may bias the comparison against SI-based methods.

In the revision, we clarify that using a more liberal SI threshold identifies a broader range of spatially modulated neurons in humans and facilitates more direct cross-species comparisons; this rationale is now made explicit in the manuscript (Lines 199–200). Under this threshold, a total of 66.9% (57.5% + 9.4%) of rodent neurons passed (Fig. 2h, bottom), whereas 10.2% (1.1% + 9.1%) of human neurons passed (Fig. 3h, bottom).

The revised section of the manuscript reads:

> Here, we use a 0.25-bit spatial information threshold, which has been applied in prior rodent study and provides a more liberal criterion, allows inclusion of a broader range of spatial tuning profiles which is important for cross-species comparisons [24]. When comparing permutation testing on both

4. In the Figure 2 legend, the description for panel e incorrectly states "same as in e."

We appreciate the reviewer for catching this typo. We have corrected the legends for Figures 2 & 3, replacing them to read "same as in d".

5. The statement that "Spatial information is most sensitive to sharp, high-contrast firing fields and is strongly modulated by the ratio of peak to baseline activity" accurately describes a property that makes SI well-suited to hippocampal place cells, though less so for neurons in other regions. However, the following point, that SI is "relatively insensitive to trial-to-trial instability and field dropout", should be balanced by noting that stability is often explicitly verified in place cell studies by comparing maps before and after experimental manipulations.

In the revised manuscript (lines 364 - 368), we now clarify that although SI itself does not capture across-trial reliability, this limitation is typically addressed by separate stability analyses, such as comparing ratemaps across session halves or odd and even trials, to ensure that place fields are consistently expressed over time. We have added this clarification and cited representative studies accordingly.

The revised section of the manuscript reads:

> Together, these simulations demonstrate that spatial information and ANOVA F-statistics capture distinct, complementary aspects of spatial tuning in single neurons. Spatial information is most sensitive to sharp, high-contrast firing fields and is strongly modulated by the ratio of peak to baseline activity, but is relatively insensitive to trial-to-trial instability and field dropout. In practice, however, this limitation is often mitigated in rodent place-cell studies by assessing stability through additional analyses which evaluate the reliability of place-related activity by examining rate maps across different groups of trials (e.g. split halves or even-odd splits) to ensure that identified fields are consistent across trials [36, 41, 49]. On the other hand, ANOVA is responsive to structured spatial variance,

6. In the section "Thresholding vs. Permutation Test and Effect of Analysis Parameters", the authors may wish to reference Grieves (2023; PMID: 38150481), which also discusses how firing rate map construction parameters (e.g., bin size, kernel type) influence place cell classification.

We have now added a citation to Grieves (2023; PMID: 38150481) in the *Results* section (Lines 243 - 248) titled *"Effects of Firing-Rate Map Construction Parameters."* This reference acknowledges prior methodological work showing that firing-rate map construction parameters (e.g., bin size, smoothing kernel) can influence spatial analyses and place-cell classification.

The revised section of the manuscript reads:

> **Effects of Firing-Rate Map Construction Parameters**
>
> Prior methodological work has shown that the parameters used to construct firing-rate maps, such as bin size and smoothing kernel, are themselves a source of variability in spatial analyses [48]. Consistent with their findings, we demonstrate that methodological choices in rate-map construction, including smoothing and spatial bin resolution, yield systematic, metric-specific effects that ultimately determine which neurons are classified as spatially tuned. Specifically, we find that applying a smoothing kernel

7. The section “Place Cell Feature Estimates” appears to repeat conclusions already stated in the preceding paragraph; consider merging or shortening these sections to improve flow.

We have condensed these sections to enhance clarity and narrative flow (Lines: 375-387).

The revised section of the manuscript reads:

Place Cell Feature Estimates

We next estimated several features of place cell activity from rat, human, and simulated neurons, including peak firing rate, average firing rate, the peak-to-average rate ratio, place field width, number of place fields, presence ratio, and even-odd correlation (Fig. **S4**, Fig. **S5**). ~~We found that spatial information (SI) correlates most strongly with the peak-to-average firing rate ratio across all datasets, indicating that SI is particularly sensitive to cells with sharply tuned, high-contrast firing fields. In contrast, ANOVA F-statistics correlate more strongly with even-odd correlation, suggesting that ANOVA captures the reliability and consistency of spatial firing patterns across repeated trials. These findings underscore that SI and ANOVA emphasize different aspects of spatial tuning and are influenced by distinct underlying neuronal firing properties (Fig. **S4**, Fig. **S5**).~~ Consistent with our simulation findings, we found that across datasets, SI correlated most strongly with the peak-to-average firing-rate ratio, whereas ANOVA correlated more with even–odd reliability, underscoring that the two metrics capture different aspects of tuning properties(Fig. **S4**, Fig. **S5**)

8. In the sentence “This organization is clarified by the feature loading vectors (Fig. 7c): the SI gradient aligns most strongly with peak-over-average firing rate (yellow) and runs opposite to place field width (cyan),” the colors do not match the figure (in Fig. 7, peak-over-average firing rate appears red and place field width magenta).

We have corrected the text so that the color descriptions align with Fig. 7.

The revised section of the manuscript reads:

average firing rate ~~(yellow),~~(red) place field width ~~(cyan),~~(magneta) even–odd correlation ~~(red),~~(yellow)
number of place fields ~~(green),~~(darkred)

9. The authors might be interested in the approach used by Savelli et al. (2017; PMID: 28084992), where they not only used a shuffling procedure to determine the significance of a metric but also used bootstrapping to better calculate the actual metric. They used this for the grid score, but it can also be easily applied to spatial information content. I think this approach could better account for trial-to-trial instability or be modified to address that issue with SI.

We thank the reviewer for bringing to our attention the bootstrapping approach of Savelli et al. (2017). The bootstrap procedure proposed by Savelli et al. offers a complementary means of quantifying uncertainty in the SI estimate itself. This approach could yield a more precise estimate of each neuron’s “true” SI value, further improving the correspondence between raw SI scores and permutation-based significance. We now note in the Discussion (Lines: 449 - 452) that incorporating bootstrapped SI estimation could be a promising direction for future methodological refinement.

The revised section of the manuscript reads:

Spatial Tuning Metric

Our analyses indicate that spatial information scores and ANOVA F-statistics emphasize different place-field features. SI primarily reflects the peak-to-average firing rate ratio: neurons with sharply localized, high-contrast fields attain high SI values, whereas neurons with elevated baseline activity more common in human recordings exhibit lower scores. SI is less responsive to trial-level variability because it is computed from the average firing rate across trials. Prior rodent studies often address this issue through additional stability measures [37, 25, 39]. Further, researchers could consider using bootstrapping to quantify how sensitive the SI estimate is to trial-to-trial variability, providing an indirect assessment of firing-field stability [40]. By contrast, ANOVA F-statistics measure how robustly

Reviewer 2:

This manuscript addresses an important methodological question in hippocampal research: how do different place cell detection methods affect cross-species comparisons? The authors systematically apply spatial information (SI) and ANOVA-based detection approaches to both rat and human datasets, supplemented by simulations with ground truth. They find that these metrics capture different aspects of spatial tuning - SI emphasises peak-to-average firing contrast while ANOVA captures trial-level consistency - and that methodological choices substantially influence which neurons are classified as place cells.

This systematic comparison of detection methods across species should be useful and important for the field, especially as human single-unit recording datasets become more prevalent.

Major Concern

1. Mischaracterisation of rodent literature undermines core message: Table 1 suggests only 3/24 rodent studies use stability measures as part of place cell classification, but this appears to significantly underrepresent standard practice. Recent rodent place cell research routinely uses some form of stability criteria to classify single units as place cells. While the authors do not elaborate on how their rodent place cell studies were selected ("we subselected a sample of studies spanning a diversity of analytical pipelines"), as a researcher in the rodent hippocampal field, I do not deem their selection to be unbiased and representative. Indeed, conducting a very brief literature search myself, I found the majority of papers I checked to use some stability criteria as part of their classification of place cells (e.g., Zhang et al 2024 Nature Comms, Blair et al 2023 eLife, Tanni et al., 2022 Current Biology, to name but a few). This selective presentation undermines a key premise that rodent researchers ignore: trial-level consistency picked up by ANOVA-based criteria. However, most crucially, this goes on to undermine one of the key messages, as defined in the Recommendations subsection: "Studies should consider the goals of their analyses and the likely properties of the place cells in their dataset to choose appropriate methods and/or consider applying multiple methods to capture different variants of putative spatial tuning." To summarise, I find the author's presentation that rodent place cell research is typically conducted by classifying place cells according to a single criterion to be overly simplistic and misrepresentative. I would suggest that a comprehensive and unbiased literature search be conducted to accurately capture the current state of the art.

We thank the reviewer for this important clarification. We agree that our original presentation in Table 1 did not sufficiently convey the extent to which rodent studies incorporate multiple place cell classification criteria, including those based on stability and reliability. Importantly, SI+stability identifies neurons with sharp, high-contrast, and consistent place fields, whereas ANOVA detects neurons whose firing varies reliably with position across trials, including broader or lower-contrast tuning that is common in rodent ventral hippocampus and in human recordings. As a result, ANOVA can be more sensitive in capturing consistent but broader and more complex spatial modulation that may support important functional roles across species, such as mixed selectivity and generalization, and that SI+stability may miss. In the revision, we have:

1. Revised the text in the Abstract (Lines 24–26), Results (Lines 364–368), and Discussion (Lines 423–424; 454–457; 509–510) to clarify that rodent studies typically rely on multiple criteria for place-cell classification. These updates provide a more balanced representation of current rodent practice and strengthen the manuscript.
2. Updated Table 1, including revising the original entries and incorporating the reviewer-suggested paper, to more accurately reflect the measurement approaches used in rodent place-cell classification.

The revised section of the manuscript reads - Abstract:

generalize to humans and uncovering principles of spatial cognition. One challenge, however, is that detection methods differ: rodent studies often rely on spatial information (SI), in conjunction with place field stability measures, whereas human studies employ analysis of variance (ANOVA) - based approaches. These methodological differences may affect the identified place cell population,

The revised section of the manuscript reads - Results:

activity, but is relatively insensitive to trial-to-trial instability and field dropout. In practice, however, this limitation is often mitigated in rodent place-cell studies by assessing stability through additional analyses which evaluate the reliability of place-related activity by examining rate maps across different groups of trials (e.g. split halves or even-odd splits) to ensure that identified fields are consistent across trials [36, 41, 49]. On the other hand, ANOVA is responsive to structured spatial variance,

The revised section of the manuscript reads - Discussion:

Discussion

Overview and Significance of the Findings

Cross-species comparisons are critical for uncovering core computational principles of hippocampal function that are either conserved or divergent across biological systems. Despite rich literatures on place cells in rodents and humans, few studies have directly compared them under comparable behavioral paradigms and analytic frameworks [21, 22, 11, 2]. Methodological conventions differ across species – rodent researchers tend to use spatial information (SI)-based methods in conjunction with other measures including place field stability(Table. 1), whereas human researchers more often employ ANOVA-based approaches (Table. 2). This lack of alignment makes it difficult to determine whether

Spatial Tuning Metric

Our analyses indicate that spatial information scores and ANOVA F-statistics emphasize different place-field features. SI primarily reflects the peak-to-average firing rate ratio: neurons with sharply localized, high-contrast fields attain high SI values, whereas neurons with elevated baseline activity more common in human recordings exhibit lower scores. SI is less responsive to trial-level variability because it is computed from the average firing rate across trials. Prior rodent studies often address this issue through additional stability measures [41, 25, 43]. Further, researchers could consider using bootstrapping to quantify how sensitive the SI estimate is to trial-to-trial variability, providing an indirect assessment of firing-field stability [50]. By contrast, ANOVA F-statistics measure how robustly

Recommendations

Together, our findings support a set of best practices for reliable and comparable place cell detection. First, while both spatial information and ANOVA effectively identify strongly tuned place cells, they differ in their treatment of borderline or noisy cases. Spatial information tends to highlight neurons with high-contrast, sharply tuned firing fields, whereas ANOVA is more likely to detect neurons with weaker but consistent modulation across trials, often characteristic of the tuning profiles observed in human recordings. Thus, the key distinction between these metrics lies not in their ability to detect canonical place cells, but in how they classify ambiguous neurons near the threshold of significance. Studies should consider the goals of their analyses and the likely properties of the place cells in their dataset to choose appropriate methods ~~and/or consider applying multiple methods to capture different variants of putative spatial tuning~~. Second, permutation-based significance testing provides greater robustness than fixed thresholds by generating neuron-specific null distributions that account for baseline variability and reduce dependence on analysis parameters such as bin size and smoothing. Together, these practices offer a more principled and reproducible framework for identifying spatially tuned neurons and enable more accurate comparisons of hippocampal spatial coding across species.

Updated Rodent Place Cell Table:

Reference	Spatial Metric	Classification Criteria	Modulation
Jung et al., 1994 [8]	SI[1]; Sparsity[2]	Place Field[3]	Place; Anatomy
Schapiro et al., 1997 [33]	N/A	Place Field[3]	Place; Cues
Wood et al., 2000 [34]	Two-Way ANOVA[4]	Place Field[3]; Parametric ANOVA main effect of spatial sector ($P < 0.05$)	Place; Trial Type
Kjelstrup et al., 2008 [35]	SI[1]	Place field[3]	Place; Anatomy
Royer et al., 2010 [36]	SI[1]; Stability[5.1]	Place field[3]	Place; Anatomy
Langston et al., 2010 [25]	SI[1]; Spatial Coherence[6]	Permutation-based SI[1] score ($P < 0.05$); Spatial Coherence < 0.14	Place; Development
Deshmukh et al., 2011 [26]	SI[1]; Stability[5.3]	SI[1] > 0.4; Permutation-based SI[1] score ($P < 0.01$); Stability > 0.71	Place; Object
Chen et al., 2013 [27]	SI[1]	Permutation-based SI[1] score ($P < 0.05$); PTD[7] $> 300\,\mu s$	Place; Visual
Roux et al., 2017 [24]	SI[1]; Stability[5.3]	SI[1] > 0.25; Permutation-based SI[1] score ($P < 0.05$)	Place; Learning
Scalpen et al., 2017 [28]	SI[1]	SI[1] > 0.25; Place Field[3]	Place; Visual
Aronov et al., 2017 [37]	SI[1]; Stability[5.1]	Permutation-based SI[1] score ($P < 0.01$)	Place; Sound
Newman et al., 2017 [38]	SI[1]	SI[1] ≥ 0.5; Place Field[3]	Place; Cholinergic Disruption
Jun et al., 2020 [39]	SI[1]	Permutation-based SI[1] score ($P < 0.05$); Place Field[3]	Place; Alzheimer's
Grieves et al., 2020 [40]	SI[1]; Stability[5.1]	SI[1] > 0.5; PTD[7] $> 250\,\mu s$	3D Place
Shuman et al., 2020 [41]	SI[1]; Stability[5.1,5.2]	Permutation-based SI[1] score and within-session stability[5.1,5.2] ($P < 0.05$)	Place
Duvelle et al., 2021 [29]	SI[1] Stability[5.3]	SI[1] > 0.5; Permutation-based SI[1] score ($P < 0.05$); PTD[7] $> 300\,\mu s$	Place; Connectivity
Harland et al., 2021 [30]	SI[1]	SI[1] > 0.5	Place; Environment Size
Jin & Lee, 2021 [10]	SI[1]; Stability[5.1]	SI > 0.5; Permutation-based SI[1] score ($P < 0.01$)	Place; Motivation
Tanni et al., 2022 [42]	Stability[5.1,5.2]	Spike Shape: PTD[8] > 0.45 ms; HW[9] > 0.1 ms; TR [10] > 0.175; Stability[5.1] > 0.25, Stability[5.2] > 0.5; Place Field[3]	Place; Perceptual Change
Levy et al., 2023 [43]	SI[1]; Spatial Coherence[6]	SI[1]; Spatial coherence[6] ($Z > 1.96$ for both)	Place
Blair et al., 2023 [44]	Stability[5.1]	Median Stability[5.1] ($P < 0.01$)	Place; Aversive Experience
Zhang et al., 2024 [45]	SI[1] Stability[5.1]	Permutation-based SI[1] score ($P < 0.05$); Stability[5.1] > 0.3	Place; Social

Table 1: Classification criteria used in rodent place-cell studies.

SI[1]: Spatial information score (bits/spike). *Sparsity[2]*: Measures how concentrated firing is across space. *Place Field[3]*: A contiguous group of adjacent spatial bins in which the average firing rate in each bin exceeds a specified threshold. *Two-Way ANOVA[4]*: A significant main effect of place was observed, but not for task-related variables or the place × task-related variable interaction. *Stability[5]*: [5.1]Split/Half-Correlation between first and second half maps. [5.2]Even/Odd-Pearson correlation from even/odd lap tuning curves.[5.3]Pixel by pixel correlation coefficient between epochs/sessions. *Spatial Coherence [6]*: Estimated as the first order spatial autocorrelation of the place field map - i.e. the mean correlation between the firing rate of each bin and the averaged firing rate up to 8 adjacent bins. *PTD[7]*: Waveform peak to trough distance. *PTD[8]*: Waveform peak-to-trough duration. *HW[9]*: Waveform peak half-width. *TR[10]*: The ratio between amplitude and trough voltage values

Minor Concerns

1. Confounding variables inadequately addressed: While the Discussion acknowledges differences between datasets (VR vs real-world, clinical constraints, anatomical sampling), these factors could explain much of the observed species differences. The interpretation that humans have "narrower" place cell distributions may reflect these experimental differences rather than biological ones.

As suggested, we revised the *'Relevance to Cross-Species Explorations' section (Lines 515-527)* to more clearly acknowledge that these factors may contribute to the comparatively narrower distribution observed in humans. The updated text explicitly states that such experimental factors could underlie part of the species differences and that these observations should not be interpreted as purely biological in nature. This clarification strengthens the framing of our cross-species comparison and better situates our findings within the broader methodological context.

Relevance to Cross-Species Explorations

Our analyses revealed both differences and meaningful overlaps in the statistical distribution of spatial tuning across species. Rat neurons exhibited a broad dynamic range on both the SI and ANOVA axes, including sharply tuned cells with high spatial contrast as well as more broadly and weakly modulated neurons. Human neurons, by contrast, clustered within a narrower range and were largely absent from the extreme high-SI and high-ANOVA region that characterizes the upper end of the rodent distribution. However, these cross-species differences may also reflect characteristics of the human dataset, which, as is normal for human datasets, is smaller than can be recorded from animal models and is obtained from patients and under constraints of recording in a hospital setting. Factors such as restricted anatomical coverage, medication status, reliance on VR-based navigation, and variability in patient engagement can influence the measurable range of spatial tuning in human place cells. Thus, the narrower dynamic range observed in the human data may be shaped by these experimental and clinical factors in addition to, rather than solely by, species-level differences. Nevertheless, subsets of

2. Limited human dataset: With only 306 neurons from 19 patients, the human data may not capture the full diversity of hippocampal responses, particularly given the clinical constraints to participant selection and medication effects. This could be represented more clearly

We agree that the limited size and clinical nature of the human dataset constrain the diversity of hippocampal responses that can be observed. While the dataset is typical in size for human intracranial studies and collected under standard clinical considerations, we now more clearly acknowledge these limitations in the *Relevance to Cross-Species Explorations* section (Lines 515 - 527), noting factors such as anatomical coverage, medication effects, and VR task demands to contribute to the observed difference between the human and rodent datasets. The revised section of the manuscript reads in (Reviewer 2, Minor Comment 1).

3. Generalisability: Focus on linear track tasks limits conclusions about 2D spatial coding.

We thank the reviewer for noting this limitation. To address it, **(i)** we now explicitly state in the Discussion (Lines 489–500) that ANOVA-based approaches have been successfully applied in prior human navigation studies using two-dimensional virtual environments; and **(ii)** we have added new 2D simulations comparing SI and ANOVA across different place-cell properties (Fig. S8). This supplemental analysis demonstrates that the patterns observed in our 1D results generalize to 2D rate maps, supporting the broader applicability of the methodological comparisons presented (see Reviewer 1, Major Comment 2).

4. Self-citation rate: while not ignoring their contribution to the field, 12/48 references from the Jacobs lab represent quite a high self-citation rate (25%). Rather than removing references, which are used appropriately, I believe the paper would benefit from a more holistic representation of the hippocampal fields, both the humans and rodents.

As suggested, we have added additional papers that contribute to comprehensive and widely used rodent place-cell identification methods, including those that combine spatial information with stability measures, as well as recent methodological references introducing new analytical frameworks. These additions provide a more holistic and representative overview of the place-cell literature, reducing the self-citation rate to 18% (12 of 68 references).

Recommendation

This manuscript makes valuable contributions to standardising place cell detection across species. However, the

characterisation of existing methods needs revision, particularly regarding stability measures in rodent studies. The authors should:

1. Conduct a more systematic literature review of rodent place cell methods (See Reviewer 2, Major Comment 1)
2. Revise claims about methodological differences between fields (See Reviewer 2, Major Comment 1).
3. More prominently discuss experimental confounds when interpreting species differences (See Reviewer 2, Minor Comments 1 & 2)
4. Consider reducing self-citations where possible (See Reviewer 2, Minor Comments 1 & 3)

With these revisions, this work would provide an important methodological resource for the field. The simulation framework and comparative analyses are valuable contributions that will help researchers make more informed choices about detection methods.

We appreciate reviewers' thoughtful feedback and believe these revisions substantially strengthen the manuscript.
