# Supplementary material for: Evaluating place cell detection methods in Rats and Humans: Implications for cross-species spatial coding
Source: PLoS Comput Biol. 2026 May 26;22(5):e1013488. doi: 10.1371/journal.pcbi.1013488 (PMC13225663; doi:10.1371/journal.pcbi.1013488)
Supplement: Attachment — Submitted filename: Response_To_Reviewers.pdf [file pcbi.1013488.s010.pdf]

Manuscript Title: Evaluating Place Cell Detection Methods in Rats and Humans – Implications for Cross-Species Spatial Coding
Manuscript Number: *PCOMPBIOL-D-25-01752*
Journal: *PLOS Computational Biology*

Dear Editor and Reviewers,

We thank the Editor and Reviewers for their thoughtful and constructive feedback and for the time and effort invested in evaluating our manuscript for the second round of revision. Below, we provide a detailed point-by-point response, with reviewer comments in **black**, our responses in **blue**, and all corresponding changes marked in **red** in the revised manuscript. We hope these revisions address all points raised by the reviewers and believe they have strengthened the manuscript.

Editor Comments:

The authors should address the few minor remaining comments from Reviewer #1 before we can proceed with publication. These final changes will be checked by the Editor - the manuscript will not be sent out for review again.

We appreciate the Editor's guidance and have revised the manuscript to address the remaining minor comments from Reviewer #1.

Response to Reviewer's Comments:

Reviewer #1: The authors have addressed my major comments. I have a couple of minor comments below, assuming the authors address these, I would recommend publication in PLOS Computational Biology.
Minor comments

1. Fig S8 needs a dedicated methods section. This can build on the existing sections, but there needs to be a description of how the 2D maps were generated and what was used to calculate the SI/F-statistic values.

We thank the reviewer for highlighting the need for a dedicated methods description for Fig. S8. In response, we have revised the Simulation Framework section (lines 642–681) to address this comment. We also clarified that the simulated place fields, G(x) in 1D linear track and G(x,y) in 2D environments, were subsequently used to compute spatial information (SI) and F-statistic values (lines 679–680).

Simulation Framework

We developed a simulation framework to generate synthetic firing rates across spatially binned locations, with tunable place field properties, in order to systematically evaluate detection metrics in both 1D linear track and 2D environments. Notably, these simulations model trial-by-trial firing rates directly, rather than simulating individual spike trains, allowing precise control over key parameters such as field width, peak rate, baseline activity, and trial-level variability (Fig. **5**).

Place Field Simulation

The activity of each neuron is modeled as a combination of a Gaussian tuning curve, a constant baseline, and additive noise on on linear (1D) and two-dimensional (2D) spatial firing field (Fig. 5a).

Place Field Peak A Gaussian curve was generated to simulate the place field of a neuron. This curve represents the neuron's firing rate as a function of spatial position along a linear track. The firing rate at position x was defined by :

$$F(x) = A \cdot \exp\left(-\frac{(x-\mu)^2}{2\sigma^2}\right) \tag{1}$$

where $F(x)$ is the firing rate at position x, A is the peak firing rate amplitude, μ is the center of the place field, and σ is the standard deviation that determines the width of the field.

For the 2D case, the place field was modeled as a 2D Gaussian field. The firing rate at position x, y was defined by:

$$F(x,y) = A \cdot \exp\left(-\frac{1}{2}\left[\left(\frac{x-\mu_x}{\sigma}\right)^2 + \left(\frac{y-\mu_y}{\sigma}\right)^2\right]\right) \tag{2}$$

where (μ_x, μ_y) defines the center of the place field, and σ determines the spatial spread equally across both dimensions. The spatial environment was discretized into equally spaced bins, with 50 bins in 1D and an 10×10 grid in 2D.

2. Fig S8 contains a number of typos, e.g. 'F-Statistc', 'Place Field Cosnstency'. The colorbar labels are also easily confused as y-axis labels.

We have corrected the typos in Fig S8 and adjusted the positions of the colorbar labels.

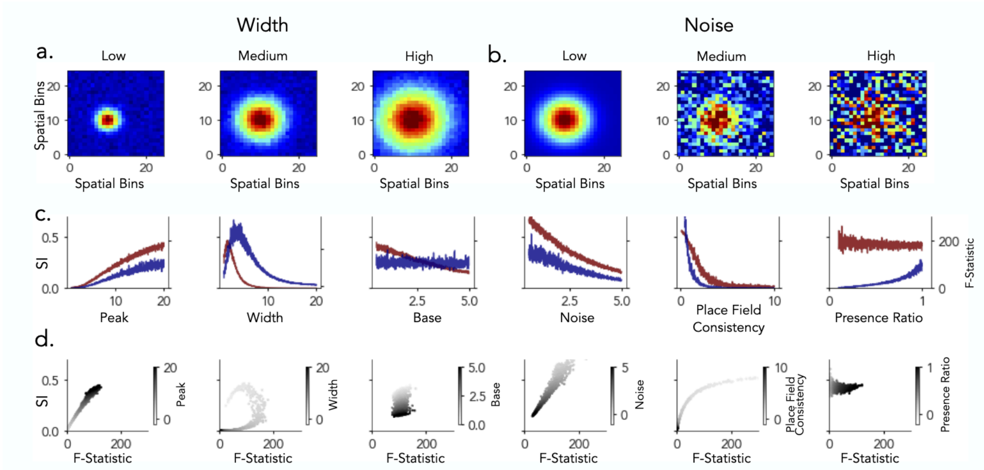

3. The Passive vs. Active Navigation section added to the discussion is informative, but the abstract will also need to be updated to reflect this limitation in interpretation. By adding, for example, "However, we cannot rule out that the passive nature of the human navigation paradigm contributed to these effects."

We agree that the passive nature of the human navigation paradigm is an important consideration for interpreting our results. Given the current length constraints of the abstract, we have clarified this point more explicitly in the Discussion under "Future Directions" (line 592). If the editor or reviewer feels that this limitation should also be explicitly stated in the abstract, we would be happy to revise it accordingly.

Addressing these questions will require accounting for differences in anatomical sampling, task design (e.g., virtual reality vs. real-world environments; passive vs. active navigation), training, and recording constraints. These efforts will be essential for studying cross-species principles of spatial coding.

4. Previous minor comment 3: The added text is useful, but it needs to be clearer that the 0.25 b/s cutoff used is lower than most rodent studies and was lowered specifically so that a larger number of human cells pass the criterion. The authors should also report how many human cells would meet the 0.5 b/s criterion.

As suggested, we now report the number of neurons that passed the 0.5 bits threshold. We also clarify in the text that this threshold represents a relatively liberal spatial tuning criterion, which captures a broader range of spatial tuning profiles, including potential conjunctive encoding. This results in a higher proportion of human neurons meeting the criterion and supports cross-species comparisons.

This point has been properly revised in the text (lines 203 - 208).

(Fig. **2**g). Here, we use a 0.25-bit spatial information threshold, as applied in prior rodent studies, to provide a more liberal inclusion criterion. This allows us to capture a broader range of spatial tuning profiles, particularly in human data, where only 0.34% of units exceed a 0.5-bit threshold, compared to 10.1% exceeding a 0.25-bit threshold. This consideration is important for cross-species comparisons [24]. When comparing permutation testing on both spatial tuning metrics, a majority of neurons

Reviewer #2: The authors have made substantial revisions that address my major and minor concerns. The revised Table 1 now includes stability measures for the majority of the rodent studies surveyed, which provides a much more accurate representation of current practice in the field. The corresponding text revisions throughout the Abstract, Results, and Discussion appropriately reflect that rodent studies typically rely on multiple criteria for place-cell classification, rather than SI alone. I am satisfied that this addresses my primary concern.

The expanded Discussion sections on cross-species confounds (lines 508–580) are a welcome addition, particularly the explicit acknowledgement that factors such as anatomical coverage, VR-based navigation, medication effects, and clinical constraints may contribute to the narrower distribution of tuning scores observed in human recordings. The new subsections on anatomical differences, virtual environments, passive vs. active navigation, and clinical constraints provide a more balanced interpretation of the species comparison.

I note that the self-citation rate has been reduced from 25% to 18% through the addition of external references, which broadens the paper's coverage of the literature.

I am satisfied that the authors have adequately addressed my concerns and consider the manuscript suitable for publication.

We thank the reviewer for their careful review and thoughtful feedback, which helped improve the manuscript.
