# Supplementary material for: Evaluating place cell detection methods in Rats and Humans: Implications for cross-species spatial coding
Source: PLoS Comput Biol. 2026 May 26;22(5):e1013488. doi: 10.1371/journal.pcbi.1013488 (PMC13225663; doi:10.1371/journal.pcbi.1013488)
Supplement: Attachment — Submitted filename: Response_to_Reviewers_auresp_3.pdf [file pcbi.1013488.s011.pdf]

Manuscript Title: Evaluating Place Cell Detection Methods in Rats and Humans – Implications for Cross-Species Spatial Coding
Manuscript Number: *PCOMPBIOL-D-25-01752*
Journal: *PLOS Computational Biology*

Dear Editor and Reviewers,

We thank the Editor and Reviewers for their thoughtful and constructive feedback and for the time and effort invested in evaluating our manuscript for the revision. Below, we provide a detailed point-by-point response, with reviewer comments in **black**, our responses in **blue**, and all corresponding changes marked in **red** in the revised manuscript. We hope these revisions address all points raised by the reviewer and believe they have strengthened the manuscript.

Response to Reviewer's Comments:

Reviewer #1: The authors have addressed many of my previous concerns; however, a fundamental issue remains regarding the abstract and the framing of the results. Specifically, the abstract fails to mention that the human and rodent datasets were collected under vastly different experimental paradigms (passive vs. active transport, and real-world vs. virtual reality).

As established in the literature (e.g. PMID: 25420065; PMID: 15390169), these variables are known to significantly modulate place cell dynamics. By omitting these differences from the abstract and the early discussion, the manuscript inadvertently implies that the observed differences are inherent species traits rather than products of the experimental design. The manuscript cannot make conclusions about cross-species differences, the methodological differences between species are too great. To prevent the data from being misconstrued or cited as a direct cross-species comparison of cell properties, it is essential that these methodological limitations are stated clearly in the abstract. I also insist that this should be acknowledged in the opening of the discussion to provide the necessary context for the results presented.

If these changes to the abstract and discussion are made I would recommend progression to publication.

We agree that the difference in experimental paradigms is an important consideration for interpreting our results, and that our contribution here is about methodological practice and does not by itself establish species-level differences .

To address this point, we have incorporated the exact changes suggested by the reviewer/editor. Specifically, we added clarifying language to both the Abstract (lines 41-45) and the beginning of the Discussion (lines 438-441). For reference, we include the relevant excerpt below.

In relation to the cited literature, the Aghajan et al paper (PMID: 25420065) on effects of virtual reality is already cited in the section of the discussion discussing this, and we have now added the Song et al (PMID:15390169) paper on active vs passive transport to the discussion section on this point.

Revision to the *Abstract (line 41-45):*

> Addressing these analytical differences allows for more direct comparisons between species, though differences in spatial tuning may still relate to variations in experimental paradigms that warrant further investigation.

Revision to the *Beginning of Discussion (line 438 - 441):*

> These observed differences may also reflect the fact that human and rodent datasets are collected under different experimental conditions, including passive versus active navigation, real-world versus virtual environments, as well as differences in anatomical sampling, rather than purely intrinsic species traits.
